# Biotopes of the intertidal zone in Clarence Island (south of the Strait of Magellan)

**DOI:** 10.3897/BDJ.11.e105726

**Published:** 2023-07-05

**Authors:** Cristian Aldea, Cristina Hernández, Leslie Novoa, Francisco Olivera, Christian Haeger, Nadja Bello

**Affiliations:** 1 Departamento de Ciencias y Recursos Naturales, Universidad de Magallanes, Punta Arenas, Chile Departamento de Ciencias y Recursos Naturales, Universidad de Magallanes Punta Arenas Chile; 2 Centro de Investigación Gaia-Antártica, Universidad de Magallanes, Punta Arenas, Chile Centro de Investigación Gaia-Antártica, Universidad de Magallanes Punta Arenas Chile; 3 GEOGAMA, Empresa de Base Científico Tecnológica, Puerto Aysén, Chile GEOGAMA, Empresa de Base Científico Tecnológica Puerto Aysén Chile

**Keywords:** barnacles, benthos, biodiversity, Darwin Core, Kawésqar National Park, mollusks, Tierra del Fuego Archipelago

## Abstract

**Background:**

The characteristics of the Strait of Magellan promote the formation of unique environments, with diverse habitats and marine organisms. This fragmentation of the landscape generates diverse little-explored ecological associations, especially in the zone of sub-Antarctic islands of the Tierra del Fuego archipelago. One way to address this lack of knowledge is through the biotope characterization methodology, with ecological units composed of the habitat and the communities associated with these environments, obtaining data and information on the dominant and incidental taxonomic groups. This is a good research model to conduct baseline studies in coastal benthic marine environments.

**New information:**

A data set in Darwin Core standard is presented of the species that make up the intertidal biotopes of Clarence Island (Tierra del Fuego Archipelago, south of the Strait of Magellan). This includes 50 identified species and the specific coordinates for each sampled location, with a total of 1400 georeferenced records. Mollusks were the most diverse taxon with 21 species, followed by algae (14 species). Sessile organisms such as the barnacles *Elminiuskingii* and *Austromegabalanuspsittacus* predominate in these ecosystems, followed by bivalve mollusks such as *Choromytiluschorus* and *Mytiluschilensis*, which together with *Nacellamagellanica* and the alga *Hildenbrandia* sp. make up more than 50% of the total records. The inclusion of biotope patterns in this study complements the information on benthic marine flora and fauna in the intertidal zone, including new records for the coast in the Clarence Island area, which is within the boundary of the Kawésqar National Park.

## Introduction

The southern Chilean administrative region denominated “Magallanes y la Antártica Chilena” (hereafter Magallanes Region) is made up of a large unique system of sub-Antarctic channels and fjords at the convergence of the Pacific and Atlantic oceans, integrating different masses of water and thus generating a unique habitat for marine life, with high levels of endemism and biodiversity (Fernández et al. 2000, Miloslavich et al. 2011). The presence of these ecological singularities means that the variations in composition, richness and structure of the rocky coastal communities are high in comparison with the rest of the temperate coasts of America (Rosenfeld et al. 2013).

The fragmentation of the landscape significantly affects the diversity of the existing benthic communities of the Magallanes Region (Valdovinos et al. 2008), where the intertidal and shallow subtidal fractions make up the coastal margin, configured mainly by portions of reduced substrate in which macroalgae play a key role as bioengineers and structurers (Jones et al. 1997). These macroalgae assemblages allow the coexistence of a large number of invertebrates, generating interactions between organisms and coastal morphology, producing the formation of biotopes.

Biotopes are part of ecosystems and refer to environments with dominant organisms and their relationship with the abiotic variables present there, being a habitat and community assemblage (Olenin and Ducrotoy 2006), encompassing organisms and abiotic components in order to describe the landscape and its ecological functionality more broadly (John et al. 2002).

Magellanic macroinvertebrates and macroalgae have developed under the influence of local conditions such as wave intensity, which can have effects on the diversity of intertidal biotopes in a fjord system (Soto et al. 2012). Some biotopes have a restricted distribution and as such are well-defined and are easily recognized as having one or more dominant organisms (John et al. 2003). An example of this is the macroalga *Macrocystispyrifera*, which recurrently characterizes its own biotope in the Magallanes Region. In the field of biotope studies, efforts have been made in coastal localities applied to intertidal and subtidal areas during the CIMAR-15 and -16 Fjords cruises (Soto et al. 2012, Soto et al. 2015, Letelier et al. 2013).

The Magallanes Region has been chosen by several expeditions for scientific purposes (Ríos et al. 2003), carrying out studies mainly of marine macroinvertebrates in the different areas of the Strait of Magellan. There have been both compilation (Aldea et al. 2020) and specific studies, the latter in the eastern end (Aldea and Rosenfeld 2011), western end (Aldea et al. 2020), channels (Letelier et al. 2013) and Tierra del Fuego (Friedlander et al. 2018), as well as the Francisco Coloane Marine Coastal Protected Area (Aldea et al. 2011). However, there are still unexplored areas, mostly adjacent islands.

Clarence Island is an important part of this ecosystem, located to the south of Brunswick Peninsula, surrounded by the Cockburn Channel, the Bárbara Channel and the Froward Pass. Administratively, Clarence Island belongs to the Magallanes Region and is within the boundaries of the former Alacalufes National Reserve (today Kawésqar National Park). Kawésqar National Park has a wide biodiversity of flora and fauna and covers the administrative provinces of Última Esperanza and Magallanes; it is one of the largest national parks in the world (Friedlander et al. 2021). This island is the habitat of macroalgae *Macrocystispyrifera* (Palacios Subiabre 2008), *Durvillaeaantarctica* (Mansilla et al. 2017) and *Mazzaellalaminarioides* (Montecinos et al. 2012), and marine invertebrates such as arthropods, polychaetes, echinoderms, nemerteans and mollusks (Palacios Subiabre 2008, Cañete et al. 2013). The inclusion of biotopes in ecological analyses of marine environments complements the functional studies of the systems, providing information on both taxonomy and associations of organisms (Olenin and Ducrotoy 2006), which when developed on Clarence Island will provide key information on the structure of this ecosystem.

The GBIF network provides data provider institutions around the world with common standards and open source tools that allow them to share information about where and when species have been recorded (GBIF: The Global Biodiversity Information Facility 2022). The databases on this platform are available to any user, contributing knowledge regarding the distribution of species and for future decisions.

Clarence Island, a little-explored ecosystem, is of great importance to obtain more information about these marine biotopes, since although research has been carried out previously, it is of a limited nature and the information is not openly available through the GBIF open access platform. This study aims to sample a large area within the southeastern limits of the Kawésqar National Park, thereby seeking to achieve a precise description of the marine biotopes present in the different intertidal and shallow subtidal strata during the summer and winter, in order to contribute to knowledge and future decision-making in the Magallanes Region.

## Project description

### Title

Determination of intertidal biotopes in the locality of Clarence Island.

### Personnel

Francisco Olivera, Christian Haeger, Nadja Bello, Javier Araneda, Cristian Serón, Victoria Riquelme, Cristina Hernández, Leslie Novoa, Cristian Aldea.

### Study area description

Clarence Island is located south of the Brunswick Peninsula and is surrounded by the Strait of Magellan, Barbara Channel, Cockburn Channel, and the narrow Pedro and Acwalisnan Channels.

### Funding

GEOGAMA Research Project (PIG-2023-MAG01).

## Sampling methods

### Study extent

The sampling was carried out as part of an exploratory study of the biodiversity of Clarence Island, located in the Magallanes Region, extending into the Chilean Fjords and Channels Ecoregion (Spalding et al. 2007). The contributions of freshwater from the ice fields of glaciers surrounding Clarence Island and the geomorphology of the area cause the studied locality to have particular and unique marine biodiversity. A part of Clarence Island called Seno Duntze, located on the southeast coast of the Island towards the Cockburn Channel and exposed to the prevailing westerly winds, was described by Palacios Subiabre (2008), who indicated that the coast has an intertidal substrate with little slope, pebble and boulder block granulometry, and sedimentary type rocks. *Macrocystispyrifera* is mentioned as the predominant algal species, along with several species of marine invertebrates (Palacios Subiabre 2008).

### Sampling description

**Figure 1.**
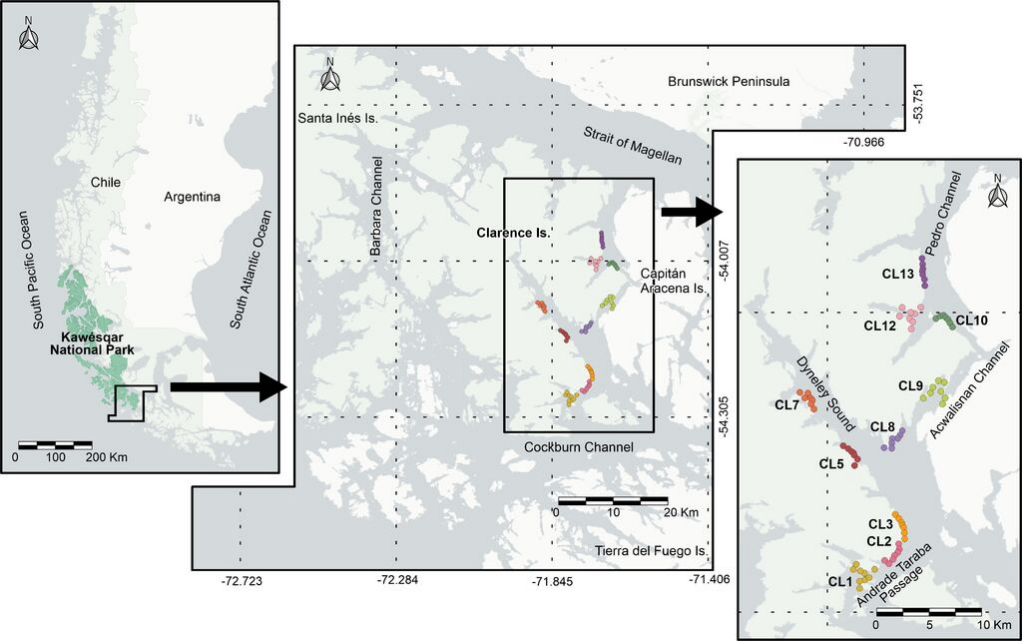
Location of the study area on the east coast of Clarence Island, which is shown circumscribed within the limits of the Kawésqar National Park (green shading). Each transect (CL1 to CL13; see Table 1) was assigned a different color.

Units of measurement in the study area were defined following a distance gradient, considering sites of interest in fjords and channels on the east coast of Clarence Island, just inside the southeastern boundary of Kawésqar National Park (Fig. 1). These units were called transects (Table 1). Stations were established in front of, around and in the immediate vicinity of each transect. Between 7 and 9 sampling units (stations; Table 1) were defined, spaced approximately every 500 meters, which gave each transect a maximum coastline prospecting distance of 4.5 kilometers.

The intertidal zone is regularly exposed to air by tidal movement; the aquatic organisms that live in these habitats are adapted to these periods. The mid-coastal zone is wide and very visible, often dominated by rocks inhabited by attached or mobile organisms which are tolerant of periodic exposure to air and depend on seawater immersion. The middle zone of the coast is preceded by a supralittoral zone, a strip of almost bare rock, although with the presence of some gastropods. Below the middle zone is the infralittoral zone, where there is a margin of dense kelp and other algae that provide shelter for flora and fauna.

The sampling was carried out with high-quality still camera photographs, which allow the identification of the species in the images. The entire bank of photographs was organized and classified by sampling season, summer and winter. All photos were taken by professionals from biological areas; special care was taken to capture the zone and representation of intertidal biotopes, following the recommendations of John et al. (2003) on intertidal biotopes.

### Quality control

The identification of taxa taken in the photograph was carried out meticulously, using the appropriate specific literature for each taxon plus comparison with samples in institutional collections. Species records and their respective geographic positions of the sites were entered into a spreadsheet, structured using the Standard Darwin Core format (Wieczorek et al. 2012) and taxonomically adjusted according to the World Register of Marine Species (WoRMS Editorial Board 2022). The data were submitted in the Integrated Publishing Toolkit, following the standards of the Global Biodiversity Information Facility (GBIF).

## Geographic coverage

### Description

The coast of the Clarence Island, south of the Strait of Magellan, in an intensive sampling area along fjords and channels in the southeast of the island, covering <1 degree of latitude.

### Coordinates

-54.2899056 and -54.0090778 Latitude; -71.8735611 and 71.6469 Longitude.

## Taxonomic coverage

### Description

All taxa were identified to the lowest possible taxonomic level. Four kingdoms, nine phyla and 13 different classes were recorded.

### Taxa included

**Table:** 

Rank	Scientific Name	Common Name
kingdom	Animalia	Animals
kingdom	Chromista	
kingdom	Fungi	Funguses
kingdom	Plantae	Plants
phylum	Arthropoda	Arthropods
phylum	Cnidaria	Cnidarians
phylum	Echinodermata	Echinoderms
phylum	Mollusca	Mollusks
phylum	Ochrophyta	Ochrophytes
phylum	Ascomycota	Ascomycetes
phylum	Bryophyta	Mosses
phylum	Chlorophyta	Green algae
phylum	Rhodophyta	Red algae
class	Thecostraca	
class	Anthozoa	Anthozoans
class	Asteroidea	Starfishes
class	Echinoidea	Sea urchins
class	Bivalvia	Bivalves
class	Gastropoda	Gastropods
class	Polyplacophora	Chitons
class	Phaeophyceae	Brown algae
class	Lecanoromycetes	Lichens
class	Bryopsida	Mosses
class	Ulvophyceae	Green algae
class	Bangiophyceae	Red algae
class	Florideophyceae	Red algae

## Temporal coverage

**Data range:** 2020-3-04 – 2020-6-30.

## Usage licence

### Usage licence

Creative Commons Public Domain Waiver (CC-Zero)

### IP rights notes

This work is licensed under a Creative Commons Attribution Non-Commercial (CC-BY-NC) 4.0 License.

## Data resources

### Data package title

Biodiversity of intertidal biotopes of Clarence Island (Tierra del Fuego Archipelago, S Chile)

### Resource link

https://doi.org/10.15468/he5828

### Alternative identifiers

http://gbif-chile.mma.gob.cl/ipt/resource?r=biodiversity-of-intertidal-biotopes-of-clarence-island-south-of-the-strait-of-magellan

### Number of data sets

1

### Data set 1.

#### Data set name

Biodiversity of intertidal biotopes of Clarence Island South of the Strait of Magellan

#### Data format

Darwin Core

#### Download URL

https://www.gbif.org/dataset/a26b09cb-37c5-4b4a-87c8-501d55bfad6e

#### Description

Fifty species were identified in the area, making up a total of 1400 georeferenced records (Aldea et al. 2023). Mollusca were the most diverse taxon, representing 42% (21 species), followed by Rhodophyta (16%, 8 species) and Chlorophyta (12%, 6 species). The most predominant taxon in terms of occurrences was Mollusca (499 records, 36%), followed by Arthropoda (366, 26%) and Rhodophyta (164, 12%). The most predominant species were the barnacles *Elminiuskingii* 203 records, (15%) and *Austromegabalanuspsittacus* (163, 12%), followed by the bivalves *Choromytiluschorus* (134, 10%) and *Mytiluschilensis* (80, 6%), the gastropod *Nacellamagellanica* (78, 6%) and the red alga *Hildenbrandia* sp. (70, 5%). Those species represent more than 50% of the records.

The following data fields of the Darwin Core standard were utilized:

**Data set 1.** 

Column label	Column description
occurrenceID	Single correlative indicator of the biological record
basisOfRecord	"Occurrence" for all records
institutionCode	The acronym in use by the institution having custody of the information referred to in the record
collectionCode	Code of the collection within the institution
catalogNumber	Correlative number
type	Records entered as "Event" or "StillImage"
language	Spanish
institutionID	The identifier for the institution having custody of the information referred to in the record
collectionID	The identifier for the collection or dataset from which the record was derived
datasetID	The code "intertidal-biotopes-clarence-island" for entire database
recordedBy	Name of the person responsible for the record
individualCount	Number of individuals recorded
occurrenceStatus	"Present"
associatedMedia	An unique identifier (URL) of the image associated with the occurrence
samplingProtocol	The sampling method for each record
eventDate	The date during which the record occurred
habitat	The intertidal zone or "Supralittoral" where each record occur
eventRemarks	The season of the event
continent	The name of the continent in which the location occurs
islandGroup	The name of the island group in which the location occurs
island	The name of the island on which the location occurs
country	The name of the country in which the location occurs
countryCode	The standard code for the country in which the location occurs
stateProvince	Location, refers to the Administrative Region of Chile
county	Location, refers to the Administrative Province of Chile
municipality	Location, refers to the Administrative Commune of Chile
locality	The specific name of the place
verbatimLocality	The original textual description of the place
verbatimElevation	The original description of the elevation (sea level) of the location
minimumElevationInMeters	The lower limit of the range of elevation (sea level)
maximumElevationInMeters	The upper limit of the range of elevation (sea level)
verbatimDepth	The original description of the depth (sea level)
minimumDepthInMeters	The lesser depth of a range of depth (sea level)
maximumDepthInMeters	The greater depth of a range of depth (sea level)
locationRemarks	The name of the transect and sampling station
verbatimCoordinates	The verbatim original coordinates of the location
verbatimLatitude	The verbatim original latitude of the location
verbatimLongitude	The verbatim original longitude of the location
verbatimCoordinateSystem	The coordinate format for the “verbatimLatitude” and “verbatimLongitude” or the “verbatimCoordinates” of the location
verbatimSRS	The spatial reference system [SRS] upon which coordinates given in “verbatimLatitude” and “verbatimLongitude” are based
decimalLatitude	The geographic latitude in decimal degrees
decimalLongitude	The geographic longitude in decimal degrees
geodeticDatum	The spatial reference system [SRS] upon which the geographic coordinates given in “decimalLatitude” and “decimalLongitude” was based
coordinateUncertaintyInMeters	The horizontal distance from the given “decimalLatitude” and “decimalLongitude” describing the smallest circle containing the whole of the location
identifiedBy	Responsible for recording the original occurrence
dateIdentified	The date-time or interval during which the identification occurred
scientificNameID	An identifier for the nomenclatural details of a scientific name
scientificName	The name of species or taxon of the occurrence record
kingdom	The scientific name of the kingdom in which the taxon is classified
phylum	The scientific name of the phylum or division in which the taxon is classified
class	The scientific name of the class in which the taxon is classified
order	The scientific name of the order in which the taxon is classified
family	The scientific name of the family in which the taxon is classified
genus	The scientific name of the genus in which the taxon is classified
specificEpithet	The name of the first or species epithet of the “scientificName”
infraspecificEpithet	The name of the lowest or terminal infraspecific epithet of the “scientificName”
taxonRank	The taxonomic rank of the most specific name in the “scientificName”
scientificNameAuthorship	The authorship information for the “scientificName” formatted according to the conventions of the applicable nomenclatural Code
vernacularName	A local common or vernacular name
verbatimIdentification	A string representing the taxonomic identification as it appeared in the original record

## Additional information

**Figure 2.**
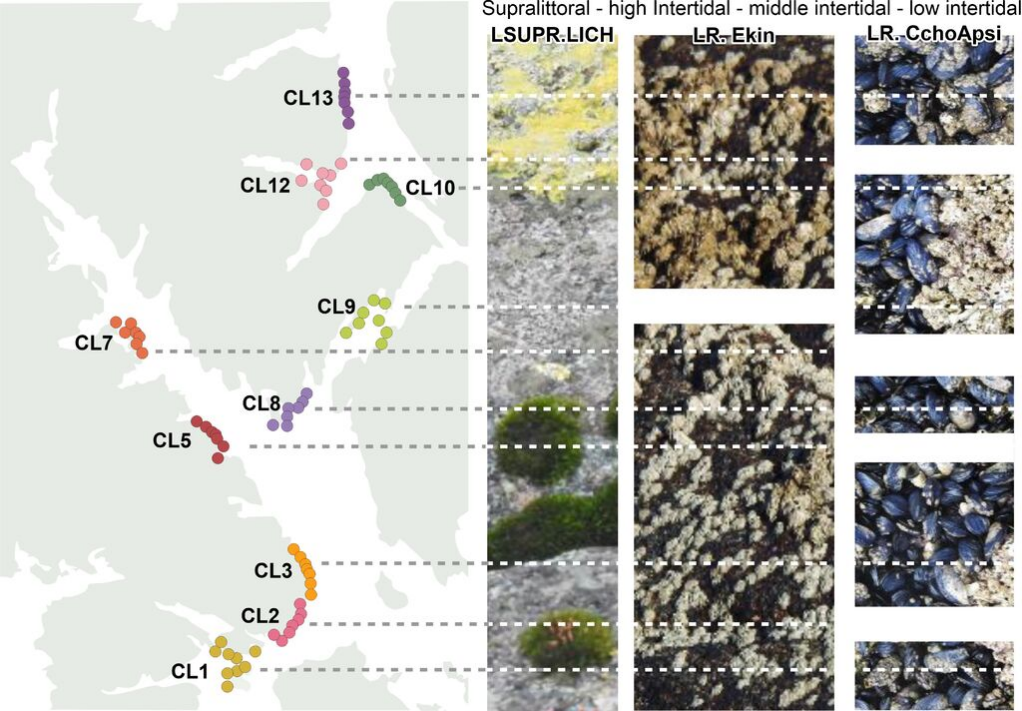
Study area showing the three most frequent biotopes in summer and winter in each intertidal zonation. Dotted lines indicate the presence of the biotope in a transect and the continuous white bands indicate absence. See Table 2 for the biotope codes.

Based on the methodology proposed by John et al. (2003) for the Aysén Region (Laguna San Rafael National Park, Estero Elefantes, Chonos Archipelago and Katalalixar Reserve), three studies related to intertidal biotopes have been carried out in the southern part of the Chilean Fjords and Channels Ecoregion (Soto et al. 2012, Soto et al. 2015, Letelier et al. 2013), all from the CIMAR-Fjords Cruises (Comité Oceanográfico Nacional 2021). In the first, 13 stations were sampled between Canal Trinidad and Canal Smyth, where 19 biotopes were identified. In the second, a sampling of 14 stations was carried out, recognizing 10 biotopes from the Strait of Magellan to the Beagle Channel. Finally, the latest biotope work carried out so far in the region, also between the Trinidad Channel and Smyth Channel, found 13 recognized biotopes in the intertidal and shallow sublittoral zones. Therefore, until now there was a lack of biotope information in remote locations such as Clarence Island, where its estuarine areas have new biotopes, based on this study. A common biotope throughout the study area is that composed of lichens and mosses (LSUPR.LICH; Fig. 2, Table 2). Other very common biotopes in the study area were those of the barnacle *Elminiuskingii* (LR. Ekin; Fig. 2, Table 2), present in almost all the stations with the exception of transect CL9, and the biotope composed of *Choromytiluschorus* and *Austromegabalanuspsittacus* (LR. CchoApsi; Fig. 2, Table 2), present in most of the stations of several transects.

### Conclusions

This report constitutes the first record of the intertidal coastal biota for the eastern coast of Clarence Island (Tierra del Fuego Archipelago) which is circumscribed in the Kawésqar National Park.

This information intends to contribute to the knowledge of the coastal ecology of fjords and channels in southern Chile, in addition to serving as a basis for establishing new species distribution records.

## Figures and Tables

**Table 1.** Location of the sampling stations for the characterization of the intertidal biotopes of Clarence Island.

**Transect**	**Station**	**Latidude / Longitude**	**Site name**
CL1	CL1-E1	54°17'23.66"S, 71°47'50.98"W	Duntze Island
CL1	CL1-E2	54°17'01.85"S, 71°47'49.50"W	Duntze Island
CL1	CL1-E3	54°16'52.35"S, 71°46'58.41"W	Duntze Island
CL1	CL1-E4	54°16'58.65"S, 71°47'21.51"W	Duntze Island
CL1	CL1-E5	54°16'27.26"S, 71°46'27.85"W	Duntze Sound
CL1	CL1-E6	54°16'37.31"S, 71°47'21.68"W	Duntze Sound
CL1	CL1-E7	54°16'30.13"S, 71°47'44.03"W	Duntze Sound
CL1	CL1-E8	54°16'09.38"S, 71°48'03.70"W	Duntze Sound
CL1	CL1-E9	54°16'24.64"S, 71°48'20.94"W	Duntze Sound
CL2	CL2-E1	54°16'00.81"S, 71°45'33.04"W	Andrade Taraba Passage
CL2	CL2-E2	54°16'11.38"S, 71°45'11.08"W	Andrade Taraba Passage
CL2	CL2-E3	54°15'58.19"S, 71°44'48.97"W	Andrade Taraba Passage
CL2	CL2-E4	54°15'46.62"S, 71°44'39.79"W	Andrade Taraba Passage
CL2	CL2-E5	54°15'37.61"S, 71°44'22.00"W	Andrade Taraba Passage
CL2	CL2-E6	54°15'27.95"S, 71°44'14.06"W	Andrade Taraba Passage
CL2	CL2-E7	54°15'11.44"S, 71°44'15.51"W	Andrade Taraba Passage
CL3	CL3-E1	54°14'56.60"S, 71°43'43.52"W	Dyneley Sound
CL3	CL3-E2	54°14'38.12"S, 71°43'43.46"W	Dyneley Sound
CL3	CL3-E3	54°14'22.08"S, 71°43'45.44"W	Dyneley Sound
CL3	CL3-E4	54°14'14.29"S, 71°43'52.44"W	Dyneley Sound
CL3	CL3-E5	54°14'05.99"S, 71°43'55.98"W	Dyneley Sound
CL3	CL3-E6	54°13'53.68"S, 71°44'09.20"W	Dyneley Sound
CL3	CL3-E7	54°13'40.25"S, 71°44'27.59"W	Dyneley Sound
CL5	CL5-E1	54°11'04.26"S, 71°47'52.21"W	Dyneley Sound
CL5	CL5-E2	54°10'44.88"S, 71°47'35.12"W	Dyneley Sound
CL5	CL5-E3	54°10'31.89"S, 71°47'52.17"W	Dyneley Sound
CL5	CL5-E4	54°10'25.14"S, 71°47'55.69"W	Dyneley Sound
CL5	CL5-E5	54°10'20.53"S, 71°48'05.86"W	Dyneley Sound
CL5	CL5-E6	54°10'11.41"S, 71°48'22.34"W	Dyneley Sound
CL5	CL5-E7	54°10'01.81"S, 71°48'47.95"W	Dyneley Sound
CL7	CL7-E1	54°08'04.76"S, 71°51'14.07"W	Dyneley Sound
CL7	CL7-E2	54°07'49.19"S, 71°51'29.04"W	Dyneley Sound
CL7	CL7-E3	54°07'37.75"S, 71°51'20.09"W	Dyneley Sound
CL7	CL7-E4	54°07'30.67"S, 71°51'29.52"W	Dyneley Sound
CL7	CL7-E5	54°07'14.95"S, 71°51'42.44"W	Dyneley Sound
CL7	CL7-E6	54°07'29.61"S, 71°52'00.37"W	Dyneley Sound
CL7	CL7-E7	54°07'11.87"S, 71°52'24.82"W	Dyneley Sound
CL8	CL8-E1	54°10'12.79"S, 71°45'12.45"W	Acwalisnan Channel
CL8	CL8-E2	54°10'15.21"S, 71°44'32.78"W	Acwalisnan Channel
CL8	CL8-E3	54°09'59.84"S, 71°44'31.32"W	Acwalisnan Channel
CL8	CL8-E4	54°09'45.82"S, 71°44'27.30"W	Acwalisnan Channel
CL8	CL8-E5	54°09'45.68"S, 71°43'59.27"W	Acwalisnan Channel
CL8	CL8-E6	54°09'36.20"S, 71°43'46.40"W	Acwalisnan Channel
CL8	CL8-E7	54°09'22.63"S, 71°43'33.71"W	Acwalisnan Channel
CL9	CL9-E1	54°07'44.43"S, 71°41'35.88"W	Acwalisnan Channel
CL9	CL9-E2	54°07'29.41"S, 71°40'58.48"W	Acwalisnan Channel
CL9	CL9-E3	54°07'12.16"S, 71°40'44.97"W	Acwalisnan Channel
CL9	CL9-E4	54°06'52.30"S, 71°40'13.77"W	Acwalisnan Channel
CL9	CL9-E5	54°06'58.14"S, 71°39'42.80"W	Acwalisnan Channel
CL9	CL9-E6	54°07'25.97"S, 71°40'02.11"W	Acwalisnan Channel
CL9	CL9-E7	54°07'46.52"S, 71°39'41.55"W	Acwalisnan Channel
CL9	CL9-E8	54°08'05.06"S, 71°39'56.57"W	Acwalisnan Channel
CL10	CL10-E1	54°04'08.37"S, 71°38'48.84"W	Pedro Channel
CL10	CL10-E2	54°03'55.66"S, 71°39'01.41"W	Pedro Channel
CL10	CL10-E3	54°03'44.97"S, 71°39'10.74"W	Pedro Channel
CL10	CL10-E4	54°03'38.14"S, 71°39'22.19"W	Pedro Channel
CL10	CL10-E5	54°03'30.93"S, 71°39'33.56"W	Pedro Channel
CL10	CL10-E6	54°03'32.95"S, 71°39'50.85"W	George Cove
CL10	CL10-E7	54°03'40.06"S, 71°40'14.30"W	George Cove
CL12	CL12-E1	54°03'02.25"S, 71°43'09.22"W	Elisa Cove
CL12	CL12-E2	54°03'18.28"S, 71°42'24.82"W	Elisa Cove
CL12	CL12-E3	54°03'22.22"S, 71°42'03.46"W	George Cove
CL12	CL12-E4	54°03'03.41"S, 71°41'31.82"W	George Cove
CL12	CL12-E5	54°04'09.61"S, 71°42'26.07"W	George Cove
CL12	CL12-E6	54°03'47.43"S, 71°42'16.60"W	Elisa Cove
CL12	CL12-E7	54°03'37.82"S, 71°42'32.42"W	Elisa Cove
CL12	CL12-E8	54°03'29.01"S, 71°43'25.27"W	Elisa Cove
CL13	CL13-E1	54°01'57.47"S, 71°41'06.11"W	Pedro Channel
CL13	CL13-E2	54°01'37.97"S, 71°41'07.06"W	Pedro Channel
CL13	CL13-E3	54°01'22.79"S, 71°41'15.48"W	Pedro Channel
CL13	CL13-E4	54°01'12.94"S, 71°41'15.94"W	Pedro Channel
CL13	CL13-E5	54°01'04.84"S, 71°41'13.28"W	Pedro Channel
CL13	CL13-E6	54°00'50.73"S, 71°41'13.46"W	Pedro Channel
CL13	CL13-E7	54°00'32.68"S, 71°41'15.54"W	Pedro Channel

**Table 2.** Biotopes present in the coastal zone of Clarence Island, zonation, period of the year and sampling stations in which they were recorded. Supralittoral zone (sup), high intertidal zone (high), middle intertidal zone (mid) and low intertidal zone (low). Summer (S), Winter (W).

**Biotope**	**Description**	**Zonation**	**Period**	**Stations**
LSUPR.LICH	lichen (genera *Caloplaca* and *Lepraria*) and mosses (mainly *Blindiamagellanica*)	sup, high	S, W	All stations of study area
LR. EkinPyro	*Elminiuskingii*, *Pyropia* sp.	high, mid	S, W	CL1 (all stations) CL3 (all stations) CL7 (all stations)
LR. Ekin	*Elminiuskingii*	high, mid	S, W	CL1 (E1, E4, E5, E6, E7, E8, E9) CL2 (all stations) CL3 (all stations) CL5 (all stations) CL7 (all stations) CL8 (all stations) CL10 (all stations) CL12 (all stations) CL13 (all stations)
LR. Hild	*Hildenbrandia* sp.	high, mid	S, W	CL5 (E1, E3, E5, E7) CL10 (all stations) CL13 (all stations)
LR. EkinHild	*Elminiuskingii* in association with *Hildenbrandia* sp.	high, mid, low	S, W	CL8 (all stations) CL9 (all stations)
LR. Apsi	*Austromegabalanuspsittacus*	low	W	CL5 (E1, E2, E3, E6, E7)
LR. Ccho	*Choromytiluschorus*	mid, low	S, W	CL7 (all stations) CL12 (all stations)
LR. CchoApsi	*Choromytiluschorus* and *Austromegabalanuspsittacus*	mid, low	S, W	CL1 (E1, E2, E3, E4, E5, E7, E8, E9) CL3 (all stations) CL8 (all stations) CL9 (E1, E2, E3 E6, E7) CL10 (all stations) CL13 (all stations)
LR. CchoApsiCoff	*Choromytiluschorus*, *Austromegabalanuspsittacus* and *Corallinaofficinalis*	mid	W	CL2 (all stations)
LR. NfasCchoUlvaMlamAutr	*Nothogeniafastigiata* in association with *Choromytiluschorus*, *Ulva* sp., *Mazzaellalaminarioides* and *Adenocystisutricularis*	low	S	CL1 (all stations)
LR. DantLspi	*Durvillaeaantarctica* and *Lessoniaspicata*	low	W	CL1 (E1, E3, E5, E6, E7) CL2 (all stations)
LR. CchoNmageFisuDant	*Choromytiluschorus* in association with *Nacella* sp., *Fissurella* sp. and *Durvillaeaantarctica*	low	S	CL2 (all stations)
LR. CchoUlvaAutr	*Choromytiluschorus* in association with *Ulva* sp. and *Adenocystisutricularis*	mid	S	CL3 (all stations)
LR. ApsiUlacAutr	*Austromegabalanuspsittacus* in association with *Ulvalactuca*, *Adenocystisutricularis* and *Durvillaeaantarctica*	low	S	CL5 (E1, E2, E5, E6, E7)
LR. CchoAutriUlacCoraCodiMlam	*Choromytiluschorus* in association with *Adenocystisutricularis*, *Ulvalactuca*, *Corallina* sp., *Codium* sp. and *Mazzaellalaminarioides*	low	S	CL5 (E3, E4)
LR. CchoApsiAutriRhiz	*Choromytiluschorus* in association with *Austramegabalanuspsittacus*, *Adenocystisutricularis* and *Rhizoclonium* sp.	low	S	CL7 (E3, E4, E5, E7) CL10 (all stations)
LR. ApsiCoffCodi	*Austromegabalanuspsittacus*, *Corallinaofficinalis* and *Codium* sp.	low	W	CL7 (all stations)
LR. Dant	*Durvillaeaantarctica*	low	W	CL8 (E1, E5)
LR. CchoMlamUlvaAutrHild	*Choromytiluschorus* in association with *Mazzaellalaminarioides*, *Ulva* sp., *Adenocystisutricularis* and *Hildenbrandia* sp.	low	S	CL9 (all stations)
LR. DantCchoApsi	*Durvillaeaantarctica*, *Choromytiluschorus* and *Austromegabalanuspsittacus*	low	W	CL9 (E1, E2, E3, E4, E5, E6, E8)
LR. CchoUlva	*Choromytiluschorus* and *Ulva* sp.	low	S	CL12 (all stations)
LR. CchoApsiPyro	*Choromytiluschorus* in association with *Austromegabalanuspsittacus*, *Pyropia* sp.	low	S	CL13 (all stations)
